# Case Report: Analysis of four cases of metastatic bladder masses after radical prostatectomy

**DOI:** 10.3389/fonc.2023.1211027

**Published:** 2023-07-28

**Authors:** Hao Wang, Dawei Xie, Jun Lu, Yifan Chu, Siqi Wang, Peng Qiao, Liyang Wu, Jianwen Wang

**Affiliations:** ^1^ Department of Urology, Beijing Chaoyang Hospital, Capital Medical University, Beijing, China; ^2^ Department of Pathology, Beijing Chaoyang Hospital, Capital Medical University, Beijing, China; ^3^ Department of Urology, Beijing Daxing District People’s Hospital, Beijiing, China

**Keywords:** prostate cancer, radical prostatectomy, bladder metastasis, prognosis, diagnosis

## Abstract

**Objective:**

The aim of this study is to investigate the clinical characteristics and diagnostic and therapeutic methods of bladder metastasis after radical prostatectomy and to improve its diagnosis and treatment.

**Methods:**

The clinical data of four patients with bladder metastasis after radical prostatectomy were retrospectively analyzed from January 2011 to December 2021. Three cases suffered from intermittent gross hematuria, and only one case was found to have an elevated prostate-specific antigen (PSA) value. Transurethral resection of bladder tumor was performed in four cases, in which one case also underwent resection of urethral mass. Three cases received endocrine therapy, one of which added intravesical instillation and radiation therapy. Another case received chemotherapy based on comprehensive treatment.

**Results:**

According to the pathological and immunohistochemical results, three cases were acinar adenocarcinoma of the prostate with Gleason score of 9, and all cases were PSA positive and negative for cytokeratin 7 (CK7) and GATA binding protein 3 (GATA-3). One case was small cell neuroendocrine carcinoma of the prostate and was positive for chromogranin A (CGA), synaptophysin (SYN), and cluster of differentiation 56 (CD56). During the follow-up period of 4 to 13 months, one case was lost to follow-up and three cases were alive.

**Conclusion:**

Bladder metastasis after radical prostatectomy is rare, and pathology combined with immunohistochemistry is the gold standard for its diagnosis. Pathological type determines its treatment. Systemic treatment is essential, and local treatment is the most palliative means. Early diagnosis and treatment is significant for better prognosis.

## 1 Introduction

Prostate cancer (PC) is one of the most common malignancies in the urological tract of middle-aged and elderly men. The prevalence is rising year by year, and the mortality rate is significant, which poses a serious risk to men’s psychological and physical wellness (1). One of the best treatments for clinically limited and locally advanced PC is radical prostatectomy (RP). Lymph nodes, bones, lungs, bladder, liver, and adrenal glands are the most common sites of metastasis in advanced PC, whereas recurrent bladder metastasis following RP is rare and has only been documented in case reports (2–4). Because of bladder masses found following RP, four patients diagnosed with PC were admitted to the Department of Urology at Beijing Chaoyang Hospital, Capital Medical University, between January 2011 and December 2021. In this article, we review and analyze the clinical and pathological information among these patients, along with a discussion on the clinical characteristics and treatment prognosis.

## 2 Clinical information and methods

### 2.1 Clinical information

Clinical data of four patients with bladder metastases following RP were reviewed at Beijing Chaoyang Hospital, Capital Medical University, from January 2011 to December 2021. Four male patients were aged 61–74 years old with an average age of 66; prostate volume size is 60.00–78.96 ml with a mean of 65.55 ml; bladder masses were discovered 6–41 months after surgery with an average of 26.75 months. All patients had different degrees of lower urinary tract symptoms such as progressive dysuria as the chief complaint, and prostate-specific antigen (PSA) was 60.00, 23.23, 537.80, and 83.00 ng/ml, respectively. The patients underwent ultrasound-guided transrectal prostate biopsy, laparoscopic RP, and regular postoperative endocrine therapy with bicalutamide in combination with leuprorelin or goserelin. The four cases were readmitted with the following complaints: two cases of intermittent painless gross hematuria with clots, one case of intermittent gross hematuria with blood dripping after urination, and one case of progressive elevation of PSA without gross hematuria, with PSA of 0.29, 0.00, 4.29, and 9.36 ng/ml, respectively (**Table 1**).

**Table 1:** Baseline characteristics of four patients.

Cases	Age	Initial admission symptoms	Initial admission PSA (ng/ml)	Readmission symptoms	Readmission PSA (ng/ml)	Time of bladder swelling found after surgery (months)	Operative method	Treatment after surgery
1	61	Dysuria	60.00	Intermittent painless gross hematuria with clots	0.29	41	TURBT	bladder instillation + endocrine therapy + external radiation therapy
2	63	Weak stream of urine	23.23	Intermittent painless gross hematuria with clots	0.00	21	TURBT+Resection of urethral tumor	Etoposide and cisplatin chemotherapy +radiotherapy
3	66	Urgency, frequency and dysuria	537.80	Intermittent gross hematuria with blood dripping	4.29	6	TURBT	CAB
4	74	Dysuria	83.00	progressive elevation of PSA without gross hematuria	9.36	39	TURBT	CAB

PSA, prostate specific antigen; TURBT, transurethral resection of bladder tumor; TURP, transurethral resection of prostate; CAB, complete androgen blockade.

**Figure 1:**
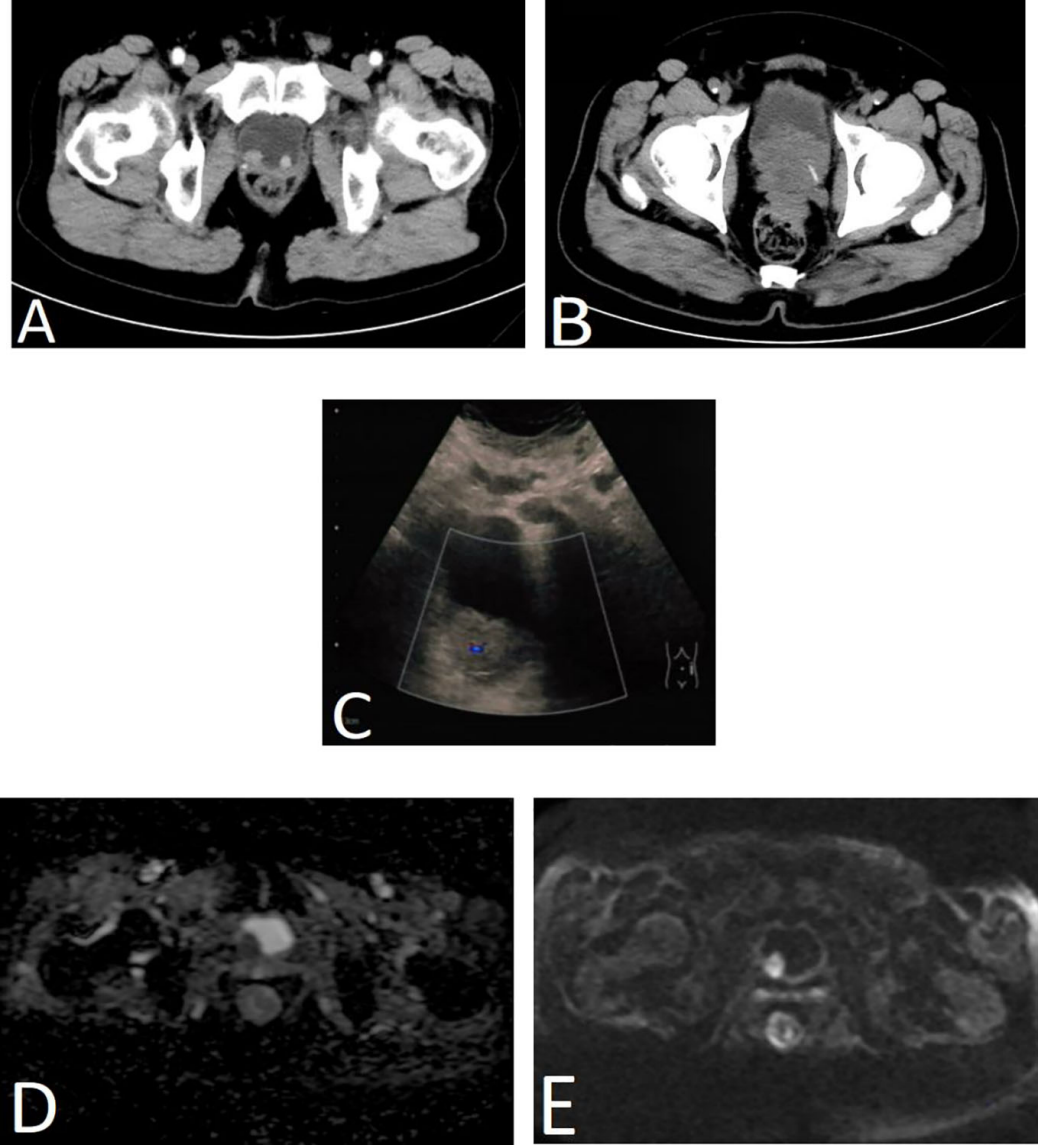
Imaging results of four patients. **(A)** Enhanced CT scan of the urinary system in case 1. Two nodule-like lesions were detected in the bladder triangle protruding into the bladder, with the right side being larger, measuring approximately 1.6 cm × 1.3 cm in diameter. **(B)** CT scan of the urinary system in case 2. Multiple hypoechoic nodules were seen in the bladder and near the bladder neck with bladder wall thickening, which was considered as recurrent prostate cancer and bladder metastasis. **(C)** Bladder ultrasound of case 3. A moderately echogenic lesion of approximately 3.9 cm × 3.3cm in size was seen in the right posterior wall of the bladder with a significant blood flow signal. **(D, E)** Prostate and bladder MRI of case 4. MRI revealed a lesion in the right posterior wall of the bladder with strong signal in DWI **(E)** and reduced ADC value **(D)**, and tumor recurrence was considered.

Ultrasound was performed in three cases, and the masses were located in the posterior bladder wall and ranged in size from 1.0 to 3.9 cm, including one case with a hypoechoic area of 5.4 cm × 5.4 cm × 6.1cm near the bladder neck. Enhanced computed tomography (CT) examination was performed in three cases, all of which showed soft tissue shadows in the bladder with varying degrees of bladder wall thickening. One case of magnetic resonance imaging (MRI) revealed iso-T1 and iso-T2 signal shadows with strong signal in diffusion-weighted imaging (DWI) and reduced apparent diffusion coefficient (ADC) value, and tumor recurrence was considered (**Figure 1**). One case was performed positron emission tomography (PET)/CT and thought to be a recurrence of PC with metastases to bladder and lymph nodes.

All specimens were formalin-fixed, paraffin-embedded, hematoxylin-eosin (HE)–stained, and immunohistochemically stained to label PSA, P504S, high–molecular weight keratin CK34βE12 (basal cells), keratin CK, GATA binding protein 3 (GATA-3), neuroendocrine markers chromogranin A (CGA), synaptophysin (SYN), and cluster of differentiation 56 (CD56).

### 2.2 Methods

All four patients were treated with surgery, in which three underwent transurethral resection of bladder tumor (TURBT) and one underwent TURBT combined with urethral mass resection. Two patients received postoperative complete androgen blockade (CAB) treatment with regular oral bicalutamide (50 mg/day) and goserelin (3.6 mg/28 day) or leuprorelin (3.75 mg/28 day) by hypodermic injection. One case received bladder instillation chemotherapy immediately after surgery and CAB treatment as well as external radiation therapy after discharge with the initial radiation dose of DT46Gy/23F and later adjusted to DT16Gy/8F. Another case received systemic therapy based on etoposide combined with cisplatin chemotherapy. The study was conducted in accordance with the Declaration of Helsinki and was approved by the Ethics Committee of Beijing Chaoyang Hospital (2020-science-299-1).

## 3 Results

**Figure 2:**
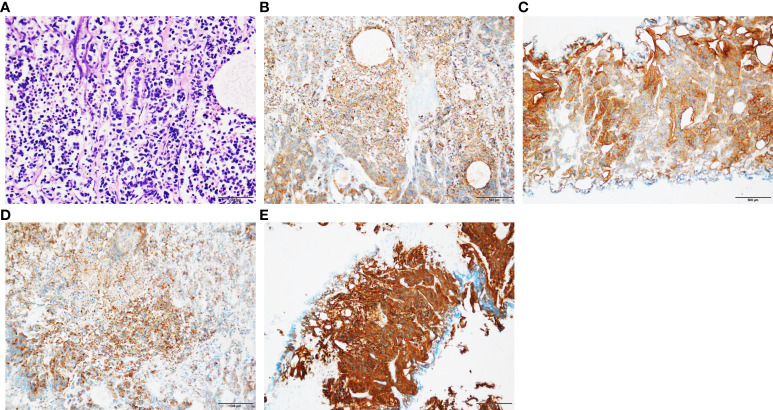
Pathological and immunohistochemical results of patients with SCNC. **(A)** Small cell carcinoma tumor cells were arranged in nest or stripe shape, with sparse cytoplasm, fine chromatin, and visible mitotic counts (HE staining; original magnification, ×100). **(B)** Positive expression of CK (immunohistochemical staining; original magnification, ×100). **(C)** Positive expression of CD56 (immunohistochemical staining; original magnification, ×100). **(D)** Positive expression of CGA (immunohistochemical staining; original magnification ×100). **(E)** Positive expression of SYN (immunohistochemical staining; original magnification, ×100).

The pathology of the bladder mass was confirmed to be of prostate origin in four patients: three cases were adenocarcinoma with Gleason score of 9, which are all positive for PSA and negative for CK7 and GATA-3; and one case was small cell neuroendocrine carcinoma of the prostate, which was not applicable to the Gleason score but was positive for CGA, SYN, and CD56. Three cases were followed up regularly, and one was lost in 2 months after surgery, with a follow-up time of 4–13 months, averaging 8 months. One received CAB treatment combined with radiotherapy after discharge, and radiation enteritis occurred during radiotherapy that was treated symptomatically. PSA was controlled at 0.01–0.02 ng/ml, and no tumor recurrence was seen in all adjuvant examinations. One case received postoperative systemic therapy based on etoposide combined with cisplatin chemotherapy, and PSA was stabilized at 0.00 ng/ml. However, chemotherapy was ineffective, and the tumor invaded the posterior bladder wall, anterior rectal wall, and pelvic floor muscles as well as developed pelvic lymph nodes, liver, and bone metastases. This patient had recurrent hematuria and urinary retention complicated by moderate to severe hydronephrosis and was in a state of bilateral nephrostomy with poor condition. Another case was treated only with CAB endocrine treatment, and PSA was controlled at around 0.02 ng/ml with no tumor recurrence observed at follow-up. The results of biopsy pathology, surgical pathology, and bladder masses pathology are shown in **Table 2**, and the pathological and immunohistochemical results about one case of small cell neuroendocrine carcinoma are shown in **Figure 2**.

**Table 2:** Summary of patient pathological results.

Case	Biopsy pathological results			Postoperative pathological results of LRP				Pathological results of bladder masses		
	Gleason score	Pathological diagnosis	Immunohistochemistry	Gleason score	Pathological diagnosis	Pathological Stage	Immunohistochemistry	Gleason score	Pathological diagnosis	Immunohistochemistry
1	4 + 5 = 9	AAP; nerve invasion	**-**	5 + 4 = 9	AAP with IDC; nerve invasion; positive surgical margins	pT3bN0M0	CK(+);P63(-)	4 + 5 = 9	AAP with DAP	CK7(-),CK20(+),PSA(+),GATA-3(-),HER2(++)
2	3 + 4 = 7	AAP; nerve invasion	P63(-),CD56(-),P504S(+),SYN(-),HCK(-),CGA(-)	4 + 5 = 9	AAP with DAP, NED; nerve invasion; positive surgical margins	pT3bN0M0	CK(+);CGA(+);SYN(+);CD56(+);P504S(partial+);P63(-);HCK(-)	**-**	SCNC	CK(+);CGA(+);SYN(+);CD56(+);P53(-)
3	4 + 5 = 9	AAP; nerve invasion	CD56(-),PSA(+),SYN(-),HCK(-),CGA(partial+)	5 + 5 = 10	AAP; nerve invasion; negative surgical margins	pT2cN0M0	P504S(+);P63(-);HCK(-);NSE(-)	5 + 4 = 9	AAP	PSA(+);CK7(-);CK20(-);GATA-3(-)
4	4 + 3 = 7	AAP with CP, BPH	P63(+);P504S(+)	4 + 5 = 9	AAP; Positive surgical margins	pT3aN0M0	CK(+);PSA(+);P504S(+);P63(-);CK34βE12(-)	4 + 5 = 9	AAP	CK7(-),CK20(+),PSA(+),GATA-3(-)

LRP, laparoscopic radical prostatectomy; AAP, acinar adenocarcinoma of prostate; CP, chronic prostatitis; BPH, benign prostatic hyperplasia; SCNC, small cell neuroendocrine carcinoma; DAP, ductal adenocarcinoma of the prostate; NED, neuroendocrine differentiation; IDC, intraductal carcinoma.

## 4 Discussion

PC is one of the most common cancers and the second most fatal diseases in males with the majority of cases developing in the peripheral zone. In China, only 33% of new cases each year are clinically limited PC, and the rest are locally advanced or metastatic patients with a much worse prognosis than cases in Europe and the United States (5). PC progresses slowly and develops distant metastasis through local infiltration or lymphatic vessels. Local lymph node metastasis (99%) and bone metastasis (84%) are the most common sites of metastasis in advanced PC; other metastasis include distant lymph nodes (10.6%), the liver and lungs (10%), as well as the brain and dura (2%), which linked with a worse survival (6).

The majority of intravesical metastases are caused by direct invasion of the bladder neck or posterior wall; cystoscopy, systematic biopsy, and immunohistochemistry of the bladder neck may be able to be used to distinguish whether it is direct infiltration or metastasis through the bloodstream. The pathology of bladder tumors is mostly uroepithelial carcinoma, and secondary bladder cancer is rare, accounting for around 2%–13% (7). Primary carcinomas of the colon/rectum, prostate, and cervix can involve the bladder directly or by hematogenous spread (8–10). Secondary bladder carcinoma is common in the bladder neck and triangle, and its clinical manifestations are mostly hematuria and dysuria. Histopathological and immunohistochemical characteristics can shed light on the tumor’s origin. All patients in this study presented with varying degrees of gross and/or microscopic hematuria, and pathological results suggested that all of the masses originated from the prostate, including three cases of adenoidal adenocarcinoma and one of small cell neuroendocrine carcinoma, which is a rare pathological type of PC with high malignancy, specific genetic and molecular alterations, as well as poor prognosis (11, 12).

The four patients in this research who developed bladder metastases after RP were high-risk PC (PSA > 20 ng/ml or Gleason score > 7/International Society of Urological Pathology (ISUP) 4/5), and the risk of lymph node metastasis can range from 15% to 40% (13). In addition, biochemical recurrence occurs in 27% to 35% of patients after RP, which is a precursor to local recurrence and distant metastasis in PC (14). There are numerous pathological factors affecting patient prognosis after RP, and studies have shown that preoperative PSA value, Gleason score, pathological stage, seminal vesicle invasion, and lymph node metastases are the key risk factors for recurrence following RP (15, 16). All four patients had a pathological Gleason score of 9 or 10, with several having adverse pathological characteristics such as seminal vesicle infiltration, nerve and muscle invasion, and positive surgical margins (PSMs), indicating high tumor aggressiveness and poor prognosis. Possible reasons for the development of metastatic bladder tumors after RP are as follows. First is direct invasion. PSM caused by surgical causes contributes to local recurrence in the cystourethral anastomosis and direct invasion of the bladder eventually. PSM has been shown to be a risk factor for biochemical recurrence, local recurrence, and metastasis (17–19). Second is lymphatic metastasis. Although pathology indicated that all cleared lymph nodes intraoperatively were negative, it could not exclude the possibility of skip metastasis and micrometastasis (20). Third is hematogenous metastases. There is a risk of prostate tumor cells entering the circulation during the procedure of RP (21). In addition, all patients in this research had intermediate to advanced PC, along with adverse pathological characteristics and a higher chance of hematogenous metastasis. Last is implantation metastasis. Surgery may cause tumor cells to be implanted in the bladder, which leads to further development of metastatic carcinoma. Aerosol produced by ultrasonic scalpel and pneumoperitoneum during laparoscopic surgery has been suggested to be associated with tumor cell dissemination and seeding (22). Postoperative pathology in three cases indicated positive apex margins and an isolated lesion that was mostly in the bladder triangle with a high probability of direct invasion. In the other case, although the margins were negative, it showed multifocal lesions, taking lymphatic and hematogenous metastasis into account. The most common site for PSM is the prostatic apex, and PSM after RP frequently necessitates adjuvant therapy, such as radiotherapy, endocrine therapy, and chemotherapy, which is beneficial in preventing and delaying biochemical and clinical recurrence as well as improving the local control rate and long-term survival. Therefore, prevention of PSM is also a key part of reducing postoperative recurrence after RP, which can avoid the possibility of clinical progression by improving surgical technique, applying neoadjuvant therapy and so on.

In terms of diagnosis, PSA value elevates for months or years after RP and reaches biochemical recurrence. It is worth noting that PSA may not be elevated in neuroendocrine PC, but neuroendocrine markers such as CGA, neuronal-specific enolase (NSE), SYN, and/or carcinoembryonic antigen (CEA) may be elevated (23). Clinical manifestations include symptoms such as hematuria, urinary frequency, and urinary tract obstruction. Imaging examinations suggest malignant features such as irregular bladder masses with moderate to high echogenicity and indistinct boundary. On the basis of the abovementioned factors, bladder metastasis of PC should be considered. Cystoscopy combined with pathological biopsy and immunohistochemical results is necessary for its correct diagnosis.

As for treatment, patients with bladder metastases and recurrence after RP are already at an advanced stage of the disease process. In principle, systemic treatment is the main method, with local treatment mostly being palliative. TURBT is an option for bladder metastases and hematuria caused by secondary malignant bladder tumors, with the majority being gross hematuria and a few being microscopic hematuria. Persistent hematuria not only can lead to anemia but also can form clots that block the urethra. Studies have shown that, for bladder tumor-derived hematuria, palliative radiotherapy can be used to alleviate hematuria and urinary tract obstruction with definite efficacy and tolerable adverse effects (24). Radiotherapy coupled with endocrine treatment can enhance patients’ quality of life and clinical efficacy in advanced recurrent metastatic PC (25). Androgen deprivation therapy (ADT) is the most widely used basic treatment for metastatic PC. Combining it with novel endocrine medicines like abiraterone and enzalutamide or chemotherapeutic medicines such as docetaxel can boost the overall outcome. Small Cell Neuroendocrine Carcinoma (SCNC) is insensitive to ADT and novel endocrine agents, and the 2020 NCCN guidelines recommend that platinum-based chemotherapy combined with etoposide is the first choice for SCNC patients (26). However, case 2 in this study was not well treated with chemotherapy; the gene test revealed breast cancer 2, early onset (BRAC-2) mutation; the overall outcome remained unsatisfactory; and prognosis was poor after the application of poly ADP-ribose polymerase inhibitor olaparib. Recently, molecular targeted agents such as Aurora A. (AURKA) kinase inhibitors, mTOR inhibitors, and anti-epidermal growth factor receptor (EGFR) pathway medicine have emerged that may benefit patient survival. Finally, for patients with advanced PC with bladder metastasis, a multidisciplinary team of urology, oncology, radiology, imaging, and pathology should be combined for joint diagnosis and treatment. In addition to systemic treatment, supportive care such as nutritional support and psychological guidance are considered, and regular condition surveillance and efficacy assessments should be performed.

In conclusion, for patients after RP, regular follow-up review should be performed to monitor serum markers such as PSA, testosterone, CGA, NSE, and SYN as well as combined with imaging examinations, clinical manifestations, etc. If bladder masses are discovered, in addition to primary bladder tumors, then secondary bladder tumors should be considered. Cystoscopy combined with histopathology and immunohistochemistry can confirm the diagnosis, and the key strategy is systemic therapy with local treatment being primarily palliative.

## Data availability statement

The raw data supporting the conclusions of this article will be made available by the authors, without undue reservation.

## Ethics statement

Written informed consent was obtained from the individual(s) for the publication of any potentially identifiable images or data included in this article.

## Author contributions

HW, JL, and JW conceived the idea and designed the study protocol. JL, LW, and PQ contributed with provision of study material or patients. HW, SW, and YF-C collected, assembled, and interpreted the data; HW and JW performed data analysis and wrote the manuscript. All authors contributed to the article and approved the final manuscript.
